# Risk of suicide ideation in comorbid substance use disorder and major depression

**DOI:** 10.1371/journal.pone.0265287

**Published:** 2022-12-07

**Authors:** Vivian N. Onaemo, Timothy O. Fawehinmi, Carl D’Arcy

**Affiliations:** 1 Division of Public Health and Preventive Medicine, Department of Community Health and Epidemiology, College of Medicine, University of Saskatchewan, Saskatoon, Saskatchewan, Canada; 2 School of Public Health, University of Saskatchewan, Saskatoon, Saskatchewan, Canada; 3 Government of Nunavut, Department of Health, Iqaluit, Nunavut, Canada; 4 Department of Psychiatry, College of Medicine, University of Saskatchewan, Saskatoon, Saskatchewan, Canada; Harvard Medical School, UNITED STATES

## Abstract

**Background:**

Suicidal behaviour is commonly associated with major depression (MD) and substance use disorders (SUDs). However, there is a paucity of research on risk for suicide ideation among individuals with comorbid SUDs and MD in the general population.

**Objectives:**

This study investigated the associated risk of suicide ideation in comorbid SUDs—cannabis use disorder (CUD), alcohol use disorder (AUD), drug use disorder (DUD) with major depressive episode (MDE) in a nationally representative sample.

**Methods:**

Multilevel logistic regression models were used to analyze the 2012 Canadian Community Health Survey- Mental Health (CCHS-MH) data. This is a cross-sectional survey of nationally representative samples of Canadians (n = 25,113) aged 15 years and older residing in the ten Canadian provinces between January and December 2012. Diagnoses of MDE, AUD, DUD, and CUD were based on a modified WHO-CIDI, derived from DSM-IV diagnostic criteria.

**Results:**

Comorbidity was found to be the strongest predictor of suicide ideation. Compared to those with no diagnosis of either a SUD or MDE, individuals with a comorbid diagnosis of AUD with MDE, CUD with MDE, or DUD with MDE were 9, 11 and 16 times more likely to have 12-month suicide ideation respectively. A diagnosis of MDE was a significant predictor of 12-month suicide ideation with about a 7-fold increased risk compared with individuals not diagnosed with either MDE or a SUD.

**Conclusion:**

Suicide is a preventable public health issue. Our study found a significantly increased risk of suicide ideation among persons who have comorbid SUD with MD. Effective integration of mental health and addictions services could mitigate the risk of suicide and contribute to better outcomes.

## Introduction

Globally, suicide is a key public health issue with an estimated 703 000 deaths by suicide annually and even more suicidal attempts [1]. In 2019, suicide was ranked the fourth leading cause of mortality in young people aged 15–29 years worldwide with more than half (58%) of global suicides occurring before the age 50 years [1]. In high-income countries such as Canada, a history of mental disorders was present in up to 90% of completed suicides [1, 2]. Since suicide is a sensitive and complex issue, a rare outcome and illegal in some countries, analytical studies on suicides are complicated and suicides are highly likely to be under-reported and misclassified [3, 4]. Suicide ideation is a major risk factor for suicide and key precursor of death by suicide [3, 5]. Approximately 3.9% of suicidal ideation is associated with increased risk for suicide death [6]. Suicidal ideations, therefore, represent surrogates for studies on suicides and associated factors [7].

Suicidal ideations often used interchangeably with suicidal thoughts or ideas, is a comprehensive term used to illustrate a range of contemplations, desires, preoccupations, or plan to end one’s life [8] while suicide attempt is self-directed behaviors leading to injury or potential for injury with direct or indirect suicidal intent usually following ideations [9]. Since suicidal ideation is associated with future suicide attempts and death by suicide [3, 10] it is, therefore, a significant clinical condition that merits investigation, particularly among individuals with substance use disorders and/or psychiatric comorbidity.

Substance Use Disorders (SUDs) which includes cannabis use disorder (CUD), alcohol use disorder (AUD), and drug use disorder (DUD) have been found to increase the risk of suicide [11–14]. In a cohort of SUD inpatients, suicidal ideation in the year prior to treatment was 28.5% and, it persisted even after treatment (2-year follow-up) in about 10.4 to 19.9% of patients [15]. It is estimated that 25–50% of all suicides are associated with SUDs and 22% attributable to AUD, meaning that every fifth suicide could have been prevented if alcohol was not consumed [2]. SUDs are the second most common cause (22.4%) for carrying out suicide in outpatient setting; the increased occurrence in inpatient setting is about two-fold [16–18]. About 40% of substance dependent individuals that seek treatment have a history of at least one suicide attempt [19]. Emerging evidence also suggests a role of cannabis on suicidal behavior [4, 20–22], and cannabis use has been found to be an independent predictor of suicide, with the frequency of use being associated with increased suicide attempts [23].

The two most common mental health disorders in North America are SUDs and major depression (MD), which often co-exist, especially among treatment-seeking patients [24–29]. A recent systematic review with meta-analysis reported a 3-fold comorbid association between CUD and major depression [30]. Suicidal behaviour is commonly associated with depression and alcohol use disorders [3] and the combination of alcohol dependence and MD is considered the leading risk factor for completed suicides [31]. While the lifetime risk of suicide is estimated to be 4% in patients with mood disorders [32], and 7% in people with alcohol dependence [33], it increases considerably with comorbidity [2]. Among adolescents, MD has been found to predict subsequent weekly cannabis use resulting in increased risk for suicidal ideation [34]. MD and alcohol dependence were respectively responsible for the first and second largest proportion of the suicide disability-adjusted life-years (DALY) that were attributable to mental health and substance use disorders in 2010 [35].

Several studies on comorbid SUD and psychiatric disorders have focused on prevalence and risk factors and associations [27, 29, 36] with very few on the impact of that comorbidity such as the associated risk of suicide. Most of the studies that have assessed the impact of comorbid SUD with psychiatric disorders used patient population samples [31, 37–39]. In particular, studies on the general Canadian population have not assessed the impact of comorbid SUD and psychiatric disorders on suicide or suicide ideation, thus, a paucity of information [40–43].

Using a nationally representative sample of Canadians to assess the impact of comorbid SUD with major depression on the risk of suicide ideation, this study aims at bridging the gap in literature and possibly shed light on suicide preventive strategies in individuals with comorbid SUD and MD.

### Objectives

This study aimed to determine the associated risk of suicide ideation in comorbid SUDs with major depressive episode (MDE) in a nationally representative sample—i.e., cannabis use disorder (CUD) with MDE, alcohol use disorder (AUD) with MDE, and drug use disorder (DUD) excluding cannabis with MDE.

## Methods

### Subjects

Subjects were participants of the Canadian Community Health Survey (CCHS), 2012 Mental Health (CCHS 2012: MH), N = 25,113, response rate = 86.3%. The CCHS 2012 MH provides a comprehensive look at mental health with respect to who is affected by specific mental health disorders, positive mental health, access to and utilization of formal and informal mental health services and support; as well as individual functionality, regardless of the presence of a mental health problem [44]. The survey included persons aged 15 years or more and resident in one of the ten Canadian provinces. Respondents living in certain remote areas, institutions, and First Nations reserves were excluded from the survey. In addition, full-time members of the Canadian Forces were not included in this survey. These excluded populations make an estimated 3% of the target national population [44].

The survey used questionnaire developed by Statistics Canada in collaboration with federal, provincial stakeholders and academic experts. The questions were design for computer-assisted interviewing (CAI) such that there was a logical flow into and out of questions based on responses, with associated specified type of answers required, online edits, and actions for non-response [44]. Respondents for the CCHS-MH 2012 were selected using a three-stage sampling design. First, geographical areas called clusters were selected for each province. Secondly, for each cluster, households or dwellings were systematically selected from a prepared dwelling list. Finally, one respondent from each household/dwelling is randomly selected after determining the number of eligible respondents in the household [44].

Data was collected under the ‘Statistics Act’ of Canada [45] and participation was voluntary, though individuals were encouraged by indicating the need for information to fill data gaps in understanding mental health [44]. Ethics approval for this secondary analysis of the CCHS, MH 2012 dataset was not sought because this analysis was done using the Public Use Microdata Files (PUMF). PUMFs are available through University Libraries across Canada. PUMFs are designed to make data widely available while still maintaining confidentiality through aggregating, capping, and completely erasing identifying variables [46]. No consent was required from participants to carry out this secondary analysis.

### Measures

#### Major depression

The World Health Organization Composite International Diagnostic Interview (WHO-CIDI) is a structured diagnostic interview based on symptoms and symptom severity associated with specific psychiatric disorders. A modified WHO-CIDI algorithm derived from the Diagnostic and Statistical Manual of Mental Disorders IV (DSM IV) was applied to the symptom data to define specific psychiatric diagnosis, in this case, Unipolar Major Depressive Episode (MDE)—lifetime and 12-month [44]. A diagnosis of MDE for the last 12 months or a lifetime required at least one episode of 2 weeks or more of persistent low mood, loss of interest or pleasure in normal activities with associated changes in sleep pattern, changes in appetite, feelings of guilt, hopelessness, impaired concentration, or suicidal thoughts [44].

#### Substance use disorders

Modified WHO-CIDI algorithms derived from DSM IV were applied to the symptom data to define substance use disorders (SUDs), that is, substance abuse and/or dependence for alcohol (alcohol use disorder -AUD), cannabis (Cannabis use disorder–CUD) and other drugs excluding cannabis (drugs use disorder- DUD) in the data. Lifetime and 12-month SUDs were defined [44]. Lifetime been referred to persons who used the substance and at some point in their lifetime met the criteria for abuse or dependence, while the 12-month SUD referred to substance use in the previous 12 months meeting the criteria for abuse or dependence [44].

#### Suicide ideation

The risk of suicide was assessed using the proxy, suicidal thoughts (ideation). Several items such as depression, thoughts of death, thoughts of committing suicide, plan to commit suicide, previous suicidal attempt and hospitalization following an attempt were used to assess for suicidal ideation. These algorithms classified respondents based on whether they ever (lifetime) thought of committing suicide or taking their own life and whether those thoughts occurred in the past 12 months [44].

#### Other measures

Other variables that were included in these analyses were: sociodemographic factors—age, sex, marital status (married, common-law, widowed, divorced or separated, single), highest level of education (less than secondary education, secondary education graduate, some post secondary education, post secondary education graduate), total household income in Canadian dollars (2012) (less than $20,000, $20,000–39,999, $40,000–59,999, $60,000–79,999, $80,000 or more), race (white, non-white), smoking status (daily, occasionally, or not at all), personal and family history of mental health disorder, history of a chronic disease and childhood traumatic events [44].

### Statistical analysis

**Fig 1:**
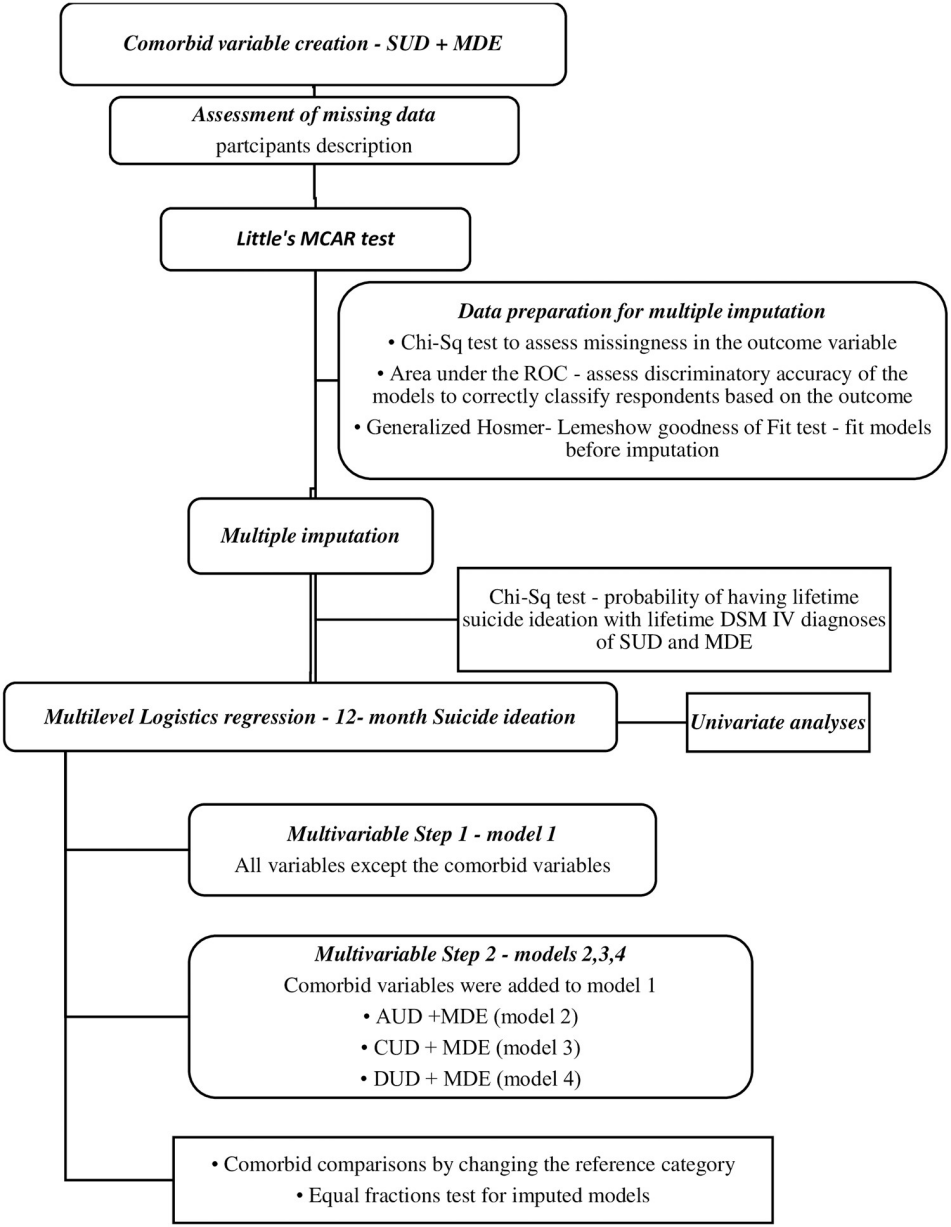
Statistical analysis flow chart lifetime DSM IV diagnoses and suicide ideation. SUD–Substance Use Disorder; MDE–Major Depressive Episode; MCAR–Missing Completely At Random; ROC–Receiver Operating Curve; DSM IV—Diagnostic and Statistical Manual of Mental Disorders IV; AUD–Alcohol Use Disorder; CUD–Cannabis Use Disorder; DUD–Drug Use Disorder (excluding cannabis); Chi-Sq–Chi Square.

A new variable, comorbid SUDs with MDE was first created by merging each SUD (alcohol, cannabis, other drugs) variable and MDE (Fig 1). For each SUD, the new variable created had four levels–no diagnosis (neither the SUD nor MDE); single diagnosis of SUD (AUD or CUD or DUD); single diagnosis of MDE; and comorbid diagnosis of SUD with MDE. Participants were described by sociodemographic characteristics and DSM IV diagnosis.

The Little’s MCAR test on Stata version 14 was used to determine if data was missing completely at random (MCAR) and a justification for a complete case analysis. Data was not missing completely at random (Little’s MCAR test: Prob > chi-square = 0.0000). Multiple imputations were then performed using chained equations following significant Chi Square associations between the independent variables and the missingness in the outcome variable, risk of suicide ideation. Prior to imputation, the area under the receiver operating curve (ROC) was used to assess the discriminatory accuracy of the models to correctly classify respondents based on the outcome. Generalized Hosmer–Lemeshow goodness of fit was used to fit the imputation models prior to imputation. After estimation with the imputed data, the models were tested for equal fraction-missing-information to check that the between-imputation and within-imputation variances were proportional.

Multilevel logistic regression models with province of residence as the group variable were used to assess the outcome–Suicide Ideation. Four models were constructed for the outcome using stepwise multivariate analyses with backward elimination of covariates. In step one, the first model was constructed for the outcome using the sociodemographic factors (age, sex, marital status, education, income, race, smoking), personal and family history of mental illness, history of childhood maltreatment and history of chronic disease. In step two, three other models were constructed by adding the comorbid substance use disorder with major depression variable (alcohol & depression- model 2, cannabis & depression–model 3 and other drugs except cannabis & depression–model 4) to the covariates listed in step one. Since the multilevel models did not allow pairwise comparison post hoc analyses for the variable ‘comorbid substance use disorder with major depression’ in the three comorbid models, comparisons were generated by changing the reference category. For each comorbid model (models 2–4), there were three analysis runs with changes to the reference category. First, the models were run for the reference category ‘no diagnosis’ of either the SUD and MDE, then for the reference category of a single diagnosis of the SUD, and finally a single diagnosis of MDE.

The odds ratio (OR) of all covariates in step 1 final model and step 2 final models were reported for the outcome. Confounding was assessed for variables eliminated from the multivariate models and re-entered into the models if present. Since the focus of the analysis was to understand the main effects and the differences between isolated substance use disorders, major depression, and the comorbidities on the risk of suicide ideation, plausible interaction terms such as gender with comorbidity or marital status with comorbidity were not assessed.

A Chi-Square test was done to depict the relationship between the DSM-IV diagnoses (isolated and comorbid) and the probability of having suicide ideation in the population. The complex sampling method of the data was accounted for by using the sampling weights provided by Statistics Canada on the survey (svy) command in Stata. All statistical analyses were carried out using STATA version 14.0.

## Results

### Participants’ characteristics

Table 1 shows weighted percentages of the sociodemographic characteristics and distribution of DSM-IV diagnoses for the target population. A third of the population were residents of the central Canadian province of Ontario and aged 25–44 years. About half of the population were married, female, were post secondary graduates with total household income of $80,000 or more. Major depressive episode (MDE), Alcohol use disorders (AUD), Cannabis use disorders (CUD), and other drugs excluding cannabis use disorder (DUD) had lifetime prevalence of 11.4%, 19%, 6.8% and 4.0% respectively while the lifetime comorbid diagnoses of MDE with AUD, MDE with CUD, MDE with DUD had prevalence of 3.2%, 1.7% and 1.3% respectively.

**Table 1:** CCHS 2012: MH participants socio-demographic characteristics and distribution of DSM-IV diagnoses.

Variables	Weighted percentages	95% CI
**Province of residence**		
Newfoundland and Labrador	1.55	1.45–1.65
Prince Edward	0.40	0.38–0.43
Nova Scotia	2.77	2.65–2.90
New Brunswick	2.15	2.05–2.26
Quebec	23.81	23.0–24.65
Ontario	38.95	38.03–39.89
Manitoba	3.43	3.20–3.68
Saskatchewan	2.82	2.67–2.97
Alberta	10.95	10.44–11.48
British Columbia	13.16	12.60–13.75
**Age**		
15 to 24 years	8.52	7.97–9.10
25 to 44 years	36.14	34.97–37.32
45 to 64 years	37.22	36.03–38.43
65 years and above	18.13	17.41–18.87
**Sex**		
Male	49.09	47.89–50.28
Female	50.91	49.72–52.11
**Marital status**		
Married	53.76	52.58–54.94
Common-law	11.89	11.14–12.69
Widowed	5.07	4.71–5.45
Divorced or Separated	8.67	7.96–9.43
Single	20.61	19.70–21.55
**Highest level of education**		
<Secondary	14.22	13.44–15.03
Secondary grad	15.70	14.09–16.55
Some post-secondary	5.96	5.40–6.57
Post-secondary grad	64.12	62.99–65.24
**Race**		
White	77.63	76.53–78.69
Non-white	22.37	21.31–23.47
**Total household income (CDN$)**		
No income or <20,000	4.16	3.77–4.59
20,000–39,999	11.84	11.22–12.48
40,000–59,999	18.15	17.29–19.04
60,000–79,999	17.75	16.85–18.68
80,000 or more	48.10	46.91–49.30
**No of types of childhood maltreatment**		
No child abuses	52.37	51.17–53.56
1–3 types of child abuses	40.28	39.11–41.47
4–6 types of child abuses	7.35	6.76–7.99
**Type of smoker**		
Daily	16.28	15.42–17.18
Occasionally	5.37	4.76–6.05
Not at all	78.35	77.31–79.36
**Family history of mental health disorder**		
Yes	39.19	38.04–40.35
No	59.85	58.69–61.00
**Personal history of mental health disorder**		
Yes	33.46	32.38–34.56
No	66.54	65.44–67.62
**12-month major depressive episode**	4.54	4.10–4.97
**Lifetime major depressive episode**	11.42	10.75–12.13
**12-month alcohol use disorder** ^1^	2.85	2.49–3.25
**Lifetime alcohol use disorder**	18.96	18.11–19.86
**12-month alcohol use disorder and major depression**	0.42	0.31–0.58
**Lifetime alcohol use disorder and major depression**	3.23	2.91–3.58
**12-month cannabis use disorder** ^2^	0.97	0.80–1.18
**Lifetime cannabis use disorder**	6.80	6.26–7.39
**12-month cannabis use disorder and major depression**	0.23	0.14–0.38
**Lifetime cannabis use disorder and major depression**	1.73	1.48–2.01
**12-month drug use disorder** ^3^	0.59	0.47–0.76
**Lifetime drug use disorder (other)**	4.03	3.64–4.47
**12-month drug use disorder and major depression**	0.25	0.17–0.38
**Lifetime drug use disorder (other) and major depression**	1.29	1.10–1.51
**12-month suicide ideation**	3.00	2.65–3.40
**Lifetime suicide ideation**	11.61	10.95–12.31

CCHS 2012: MH—Canadian Community Health Survey (CCHS), 2012: Mental Health Component

< Secondary- less than secondary education; Secondary grad- secondary education graduate; Some post sec–some post secondary education; Post-secondary grad–post secondary education graduate

^1^Alcohol abuse or dependence;

^2^Cannabis abuse or dependence;

^3^Drugs (excluding cannabis) abuse or dependence

### Comorbid diagnosis and suicide ideation

The lifetime and 12-month prevalence of suicide ideation were 11.6% and 3.0% respectively (Table 1). Table 2 shows factors associated with 12-month suicidal ideation. The odds of having a suicidal thought in the last 12 months is increased among individuals with a history of chronic health condition, childhood maltreatment, family and personal history of mental illness. Older age, being married or living in common-law, and being a graduate (secondary or post secondary) were protective from 12-month suicidal ideation.

**Table 2:** Participants sociodemographic factors associated with 12-month suicide ideation (model 1).

Crude OR (95% CI)		^a^Adjusted	
		OR (95% CI)	Statistic (p-value)
**Age**			<0.0001
15 to 24 years	1	1	
25 to 44 years	**0.63** (0.50–0.80)**	**0.61** (0.50–0.75)**	
45 to 64 years	**0.47** (0.42–0.53)**	**0.39** (0.32–0.49)**	
65 years and above	**0.22** (0.18–0.27)**	**0.21** (0.16–0.27)**	
**Sex**			
Male	1	1	
Female	1.08 (0.96–1.22)	1.08 (0.92–1.27)	
**Marital Status**			0.0003
Single	1	1	
Married	**0.37** (0.33–0.41)**	**0.72** (0.64–0.81)**	
Common-law	0.51 (0.26–1.00)	**0.56** (0.35–0.89)*	
Widowed	**0.36** (0.22–0.60)**	0.96 (0.56–1.64)	
Divorced or Separated	0.89 (0.73–1.08)	1.02 (0.82–1.28)	
**Educational status**			0.05
<secondary	1	1	
Secondary grad	**0.67** (0.52–0.87)**	**0.64** (0.46–0.90)*	
Some post sec	0.95 (0.65–1.39)	0.60 (0.34–1.04)	
Post-secondary grad	**0.58** (0.50–0.68)**	**0.69** (0.52–0.91)*	
**Race**			
White	1	1	
Non-white	1.13 (0.88–1.45)	**1.34** (1.12–1.61)**	
**Total Household Income ($)**			<0.0001
No income or <20,000	1	1	
20,000–39,999	0.69 (0.44–1.10)	0.95 (0.64–1.42)	
40,000–59,999	**0.48** (0.29–0.80)*	0.81 (0.52–1.27)	
60,000–79,999	**0.44** (0.29–0.65)**	0.72 (0.46–1.13)	
80,000 or more	**0.30** (0.22–0.40)**	**0.51** (0.40–0.67)**	
**Type of smoker**			0.04
Not at all	1	1	
Daily	**2.38** (2.09–2.72)**	**1.19** (1.01–1.40)*	
Occasionally	**2.36** (1.48–3.76)**	1.43 (0.96–2.13)	
**Types of childhood maltreatment**			<0.0001
None	1	1	
1–3 types	**2.66** (1.94–3.64)**	**1.80** (1.21–2.68)*	
4–6 types	**8.08** (6.08–10.77)**	**3.05** (2.29–4.06)**	
**History of chronic disease**			
Yes	**3.05** (2.60–3.58)**	**2.47** (2.02–3.03)**	
No	1	1	
**Family history of mental health disorder**	**2.70** (2.50–2.91)**	**1.62** (1.38–1.89)**	
Yes	1	1	
No			
**Personal history of mental illness**			
**Yes**	**7.06** (5.61–8.88)**	**4.41** (3.42–5.69)**	
**No**	1	1	

^a^—adjusted in a multivariate model; Significant values are marked in bold print

* p-value < = 0.05

** p-value <0.01

Variation explained by the Province of residence is 0.001% (p-value = 0.7)

< Secondary- less than secondary education; Secondary grad- secondary education graduate; Some post sec–some post secondary education; Post-secondary grad–post secondary education graduate

Compared to those with no diagnosis (Table 3) of either the SUD or MDE, individuals with comorbid diagnosis of AUD with MDE, CUD with MDE, DUD with MDE were respectively 9.0, 11.3 and 16.2 times more likely to have suicide ideation in the last 12 months (OR 95% CI—**9.0,** 5.6–14.5**; 11.3,** 7.6–16.8**; 16.2,** 11.1–23.6). Individuals with comorbid diagnosis of AUD with MDE, CUD with MDE and DUD with MDE had 5, 8 and 6-fold increase respectively, in the odds of having 12-month suicide ideation when compared with individuals with only the SUD diagnosis (OR 95% CI—**5.6,** 4.3–7.3**; 8.1,** 5.0–13.1**; 6.6,** 5.1–8.6). A diagnosis of MDE only had about 7-fold increase in the odds of suicide ideation in the last 12 months when compared with no diagnosis of either MDE or a SUD. MDE also had 3, 4 and 5-fold increase in the risk of 12-month suicide ideation when compared to individuals with a diagnosis of DUD, AUD, and CUD (OR 95% CI—**2.9,** 2.2–3.9**; 4.1,** 2.1–7.9**; 5.2,** 3.8–7.1) respectively. A single diagnosis of DUD only, gave a 2-fold increase in the odds of 12-month suicide ideation compared to individuals with no diagnosis of either DUD or MDE.

**Table 3:** Lifetime DSM-IV diagnoses and the risk of 12-month suicide ideation (models 2–4).

OR (95%CI)			
	*Ref = ND*	*Ref = SUD*	*Ref = MD*
**Alcohol and major depression (model 2)**			
ND	1	0.62 (0.38–1.02)	**0.15** (0.11–0.22)**
AUD only	1.62 (0.98–2.66)	1	**0.25** (0.13–0.48)**
MDE only	**6.59** (4.58–9.47)**	**4.08** (2.10–7.93)**	1
AUD & MDE	**9.02** (5.61–14.49)**	**5.58** (4.28–7.27)**	1.37 (0.80–2.35)
**Cannabis and major depression (model 3)**			
ND	1	0.72 (0.51–1.01)	**0.14** (0.09–0.22)**
CUD only	1.39 (0.99–1.96)	1	**0.19** (0.14–0.27)**
MDE only	**7.17** (4.49–11.46)**	**5.16** (3.75–7.09)**	1
CUD &MDE	**11.27** (7.57–16.78)**	**8.11** (5.01–13.12)**	1.57 (0.86–2.88)
**Other drugs (excluding cannabis) and major depression (model 4)**			
ND	1	**0.41** (0.28–0.60)**	**0.14** (0.09–0.21)**
DUD only	**2.44** (1.66–3.59)**	1	**0.34** (0.26–0.46)**
MDE only	**7.13** (4.76–10.68)**	**2.92** (2.18–3.91)**	1
DUD &MDE	**16.19** (11.10–23.63)**	**6.63** (5.10–8.64)**	**2.28** (1.47–3.53)**

Significant values are marked in bold print

* p-value < = 0.05

** p-value <0.01

^1^—reference category

Models were adjusted for age, gender, marital status, highest level of education, race, smoking status, types of childhood maltreatment, and family history of mental health disorder in multivariate analyses

Variation explained by the Province of residence: model 1 = 0.01% (p-value = 0.6); model 2 = 0.01% (p-value = 0.4); model 3 = 0.01% (p-value = 0.4)

ND–no diagnosis of either the SUD or MDE; AUD—Alcohol use disorders is defined DSM-IV Alcohol Abuse /or Dependence diagnoses.; CUD—Cannabis use disorders is defined DSM-IV Cannabis Abuse /or Dependence diagnoses.; DUD—Drug use disorders is defined as DSM-IV diagnoses of drug abuse/or dependence diagnoses on opiates, sedatives, tranquilizers, amphetamines hallucinogens, heroin, cocaine, inhalants, and/or other drug except cannabis.; MDE—Major Depression is defined DSM-IV diagnosis of major depressive episode. SUD–AUD (model 1), CUD (model 2) and DUD (model 3)

**Fig 2:**
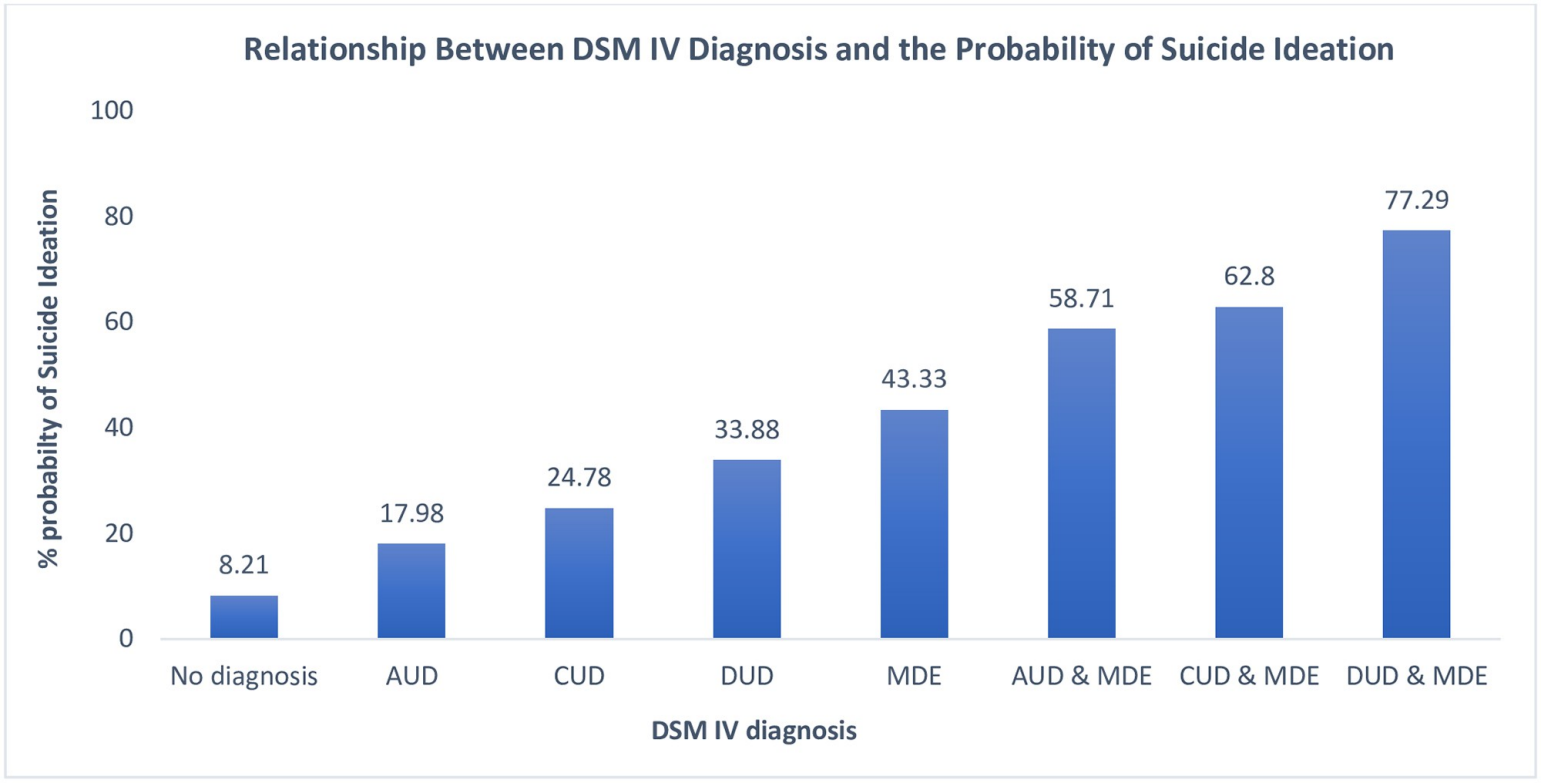
Relationship between lifetime DSM-IV diagnosis and the probability of lifetime suicide ideation. AUD—Alcohol use disorder, defined DSM-IV Alcohol Abuse /or Dependence diagnoses.; CUD—Cannabis use disorder, defined DSM-IV Cannabis Abuse /or Dependence diagnoses; DUD—Drug use disorder, defined as DSM-IV diagnoses of drug abuse/or dependence diagnoses on opiates, sedatives, tranquilizers, amphetamines hallucinogens, heroin, cocaine, inhalants, and/or other drug except cannabis.; MDE—Major depressive episode, defined DSM-IV diagnosis of major depressive episode.

The relationship between DSM-IV diagnosis and the probability of a lifetime suicide ideation in the population is shown in Fig 2. The probability of lifetime suicide ideation was <10% in individuals with no diagnosis of either SUD (AUD, CUD or DUD) or MDE. There was 18–34% probability of a lifetime suicide ideation with isolated diagnosis of a SUD only. With a diagnosis of comorbid SUD with MDE, the lifetime probability of suicide ideation ranged from 58–78%.

## Discussion

### Comorbid SUD with MDE and risk of suicide ideation

The past 12-month prevalence of AUD, CUD, DUD, and MDE were 2.9%, 0.97%, 0.6% and 4.5% respectively. Our study found that the strongest and most consistent risk factor for 12-month suicide ideation was comorbidity. When compared with individuals with no diagnosis of either SUD or MDE, the odds of having suicide ideation was associated with comorbid SUDs with MDE. This risk was much higher than the risk associated with that of a sole diagnosis of MDE. Comorbid SUDs with MDE significantly increased the odds of 12 months suicide ideation when also compared with the sole diagnosis of AUD, CUD, or DUD. Findings from our study showed a *gradual increase* in the probability of having suicidal thoughts in a lifetime from ‘no diagnosis’ of either SUD or MDE to comorbid diagnosis of SUD with MDE in the general non-institutionalized Canadian population.

The prevalence of MDE in our study, which is about twice that of AUD, is consistent with the findings from a previous survey of the Canadian population [47]. Compared to our Canadian study, in the United States (USA) a much higher prevalence has been found for depression [48], alcohol use disorder [28, 49], cannabis and drugs use disorder [29, 49]. Prevalence of comorbid diagnosis of SUD with MDE in our study was much lower than those reported in the US [49]. These variations in the prevalence of SUD, MDE and comorbid SUD with MDE could be due to different population characteristics, social and cultural characteristics, research methodologies, and diagnostic criteria.

The increased risk of 12-month suicide ideation found in our study were consistent with previous findings showing a disproportionate increase in suicide ideation with comorbid SUD with MDE and a sole diagnosis of MDE or DUD [36, 47, 50–52]; and supports findings of increased risk of suicide attempts in SUD co-existing with psychiatric disorders particularly depression [37, 53, 54]. This study also supports evidence that demonstrated AUDs and major depression as the most diagnosed major pathological disorders among persons who commit suicide [55–57]. In addition, there was two to five-fold increase in the odds of suicide ideation in individuals with MDE when compared with those with a diagnosis of a SUD only. This support previous findings of a study among patients discharged from a US Veterans Health Administration facility which found that patients who had a primary diagnosis of MD without a secondary AUD, had substantially increased prospective risk of suicide attempts [52].

The associated increased risk of suicide ideation/ suicidality in comorbid SUDs with MDE could be explained by the complicated interactions between SUDs and MDE, whereby, depression mediates the effect of SUD on suicide or SUD being the consequence of MDE [58]. Another possible explanation for this relationship is an underlying shared vulnerability to SUD and major depression, where common environmental and/or genetic factors predisposes an individual to developing mental health problems including comorbidity and consequences such as suicidality, through impaired psychosocial adjustment [59–62]. Studies also suggest that suicidal behaviour, aggression, and alcoholism have been linked to abnormally low serotonergic function [63, 64]; and this has been proposed to mediate an individual’s genetic and developmental predispositions [65]. In addition, studies on humans and animals alike have shown that increased uninhibited psychopathology, substance abuse, and impulsive aggression were secondary to serotonin abnormalities [64, 66, 67]. Substance use disorder have been shown to be associated with the recurrence and persistence of major depression [68] while protracted and untreated depression has been linked to considerable negative outcomes such as increased risk for chronicity or relapse, higher risk for comorbidity and impairments in functioning [69]. It is possible that our findings of increased risk of suicide ideation with comorbid SUD with MDE, and isolated MDE may be accounted for by a delay in treatment or non-response to depression treatment.

The significant association of DUD with suicide ideation observed in our study corroborates the earlier studies [4, 33, 70–72]. However, in contrast to previous reports [4, 22, 50, 73] our study did not find an increased risk of suicide ideation in AUD and CUD only, after controlling for sociodemographic factors, tobacco smoking status and family history of mental illness. Tobacco smoking has been proven in a meta-analysis to be associated with the risk of suicidal behaviour [74], therefore confounding the relationship between SUD and major depression. These negative findings could also be attributed to the DSM IV substance use disorder criteria, which includes the presence of DSM IV substance abuse or DSM IV substance dependence without an indication of severity as seen in DSM V. On the other hand, this finding substantiates previous research of no association in low to moderate severity of cannabis use [22].

### Strengths and limitations

A strength of this analysis is that it was based on a nationally representative sample of the Canadian population which provides insight into these comorbidities among individuals irrespective of their health-seeking behaviors. Another strong point was that the diagnoses of SUD and MDE were derived from DSM IV criteria using a structured diagnostic interview and algorithm (WHO-CIDI). This study included different comparison groups of SUD and MDE highlighting the actual effects of comorbid associations. Missing values were accounted for using multiple imputations and the complex data structure was accounted for with multilevel random effects models and survey weights, enabling generalizability.

Limitations of this study include the cross-sectional study design which does not allow for causal inference. Individuals living on reserves and other Aboriginal settlements, in institutions and full-time members of the Canadian Forces which make up about 3% of the total population were excluded from the data. Therefore, our analysis may have underestimated the true strength of associations of interest. Assessments were based on self report using computer assisted interviewing, therefore, recall and misclassification biases are possible especially with different time frames (12 months and lifetime) of SUD, MDE and suicide ideation being captured. In addition, polysubstance use or multiple diagnosis with other mental health disorders cannot be deduced from the data, therefore, there is the possibility that these individuals may have been included in our models. However, this was addressed using multivariable models to account for confounding due to history of a mental illness. The comorbid comparisons were based on a 12-month suicide ideation, and this may have underestimated the true effects. Our study was based on the available data, ‘suicide ideation’, which is a weak proxy to suicide compared to suicidal attempt. Generalization should be with caution. Further studies would be required to explore causality, comorbid associations on lifetime suicide ideation, suicidal attempts, and polysubstance comorbidity with major depression.

## Conclusion and public health implications

This study provides further evidence of associated risk of suicide ideation among persons who have comorbid alcohol use disorders, cannabis use disorders, drug use disorders with major depression. Suicide ideation among individuals with MD and comorbid SUDs tend to present with more severe mood symptoms, higher risk of suicide attempts, poor functioning, more psychiatric comorbidities, and increased mortality [75, 76]. Despite this knowledge, public health mental services are seriously underutilized by individuals with suicidal thoughts [77], and their suicide ideations usually do not come to the attention of clinicians [78]. In addition, evidence suggests increased suicides among clients with AUD or SUDs who have associated high disengagement from services in the form of skipping scheduled appointments and leaving the hospital after declining further treatment [79].

Effective integration of mental health and addictions (MHA) services with a collaborative approach that includes a multi-disciplinary team of MHA treatment providers, healthcare practitioners, family members, and community resources are essential to the effective engagement, development of preventative strategies and potentially overcoming a number of structural and behavioural impediments that support suicide ideation. This may contribute to better treatment outcomes.
